# The association of non-HDL-C, NHHR, RC, and RCII with coronary artery stenosis severity in patients with acute coronary syndrome combined with cardiometabolic multimorbidity

**DOI:** 10.3389/fendo.2025.1701364

**Published:** 2025-12-04

**Authors:** Peng Zhang, Degang Mo, Chao Dong, Wenhua Zeng, Nuo Li, Jiani Wu, Guoan Wang, Hongyan Dai

**Affiliations:** 1 Qingdao Medical College, Qingdao University, Qingdao, China; 2 School of Clinical Medicine, Shandong Second Medical University, Weifang, China; 3 Department of Cardiology, Qingdao Municipal Hospital, Qingdao, China

**Keywords:** acute coronary syndrome, multimorbidity, coronary artery stenosis, Gensini score, non-high-density lipoprotein cholesterol to high-density lipoprotein cholesterol ratio, remnant cholesterol inflammatory index

## Abstract

**Background:**

Coronary heart disease, particularly acute coronary syndrome (ACS), is a significant public health concern, and its progression is expedited when combined with cardiometabolic multimorbidity (CMM). The non-high-density lipoprotein cholesterol to high-density lipoprotein cholesterol ratio (NHHR) and remnant cholesterol inflammatory index (RCII) are novel lipid composite indices generated from non-high-density lipoprotein cholesterol (non-HDL-C) and remnant cholesterol (RC). This study examined the association between the aforementioned four indices and the degree of coronary artery stenosis (CAS) in individuals with ACS and CMM.

**Methods:**

This retrospective cross-sectional study encompassed 298 patients diagnosed with ACS and CMM who underwent coronary angiography while hospitalized. Logistic regression models and restricted cubic spline analyses were used to investigate the association between non-HDL-C, NHHR, RC, and RCII with CAS. Two-stage logistic regression models were used to analyze threshold effects, while receiver operating characteristic analysis was conducted to test the predictive capability for severe CAS. Subgroup analyses were performed to evaluate risk among different demographic groups.

**Results:**

Among the 298 participants, 150 (50.34%) had severe CAS. In multivariate logistic regression models, non-HDL-C, NHHR, RC, and RCII, when assessed per standard deviation, all exhibited a significant association with severe CAS. Among these, RCII had the strongest association with severe CAS (OR: 2.78, 95% CI 1.30-5.94), followed by NHHR (OR: 1.96, 95% CI 1.43-2.68). RCS analysis revealed a nonlinear relationship between RCII and severe CAS, with threshold effect analysis identifying an inflection point at 0.64. ROC analysis indicated that NHHR exhibited the greatest predictive capability, followed by RCII. The interaction test indicated no statistically significant difference in the association between the aforementioned four indices and CAS across subgroups.

**Conclusions:**

Non-HDL-C, NHHR, RC, and RCII all showed a strong association with CAS in patients with ACS combined with CMM. RCII exhibited a nonlinear association with severe CAS, featuring an inflection point at 0.64.

## 1 Introduction

Coronary heart disease, particularly acute coronary syndrome (ACS), is one of the leading causes of global mortality (1, 2). The burden of ACS is escalating annually due to the aging population and detrimental health behaviors (3). Cardiometabolic multimorbidity (CMM), including hypertension, diabetes, and stroke, is intricately associated with ACS (4). CMM accelerates the development of atherosclerosis via shared pathways, including chronic inflammation, insulin resistance, and oxidative stress, ultimately aggravating coronary artery stenosis (CAS) significantly (5–7). This increases patients’ overall cardiovascular risk and negatively affects prognosis. Despite low-density lipoprotein cholesterol (LDL-C) being traditionally viewed as a fundamental metric for cholesterol management and atherosclerosis evaluation (8), its capacity to explain the course of CAS in individuals with ACS and CMM remains limited.

In recent years, non-high-density lipoprotein cholesterol (non-HDL-C) and remnant cholesterol (RC) have received considerable focus as unconventional markers of atherosclerosis (9, 10). Non-HDL-C is determined by subtracting high-density lipoprotein cholesterol (HDL-C) from total cholesterol (TC) (11). RC is determined by subtracting HDL-C and LDL-C from TC (12). These two types of lipids encompass various triglyceride-rich lipoproteins (TRL) and their remnants, which accumulate in the vascular endothelium and facilitate the development of atherosclerosis (13–15). Simultaneously, within the context of CMM, systemic chronic inflammation and glucose metabolism disorders are interconnected, together amplifying the atherogenic impact of non-HDL-C and RC (16, 17). The non-high-density lipoprotein cholesterol to high-density lipoprotein cholesterol ratio (NHHR) and the remnant cholesterol inflammatory index (RCII), generated from non-HDL-C and RC, combine lipid metabolism and chronic inflammation. Growing research indicates that NHHR and RCII are becoming increasingly significant in evaluating the risk and prognosis of cardiovascular and cerebrovascular disorders (18–20). Consequently, these indices may offer enhanced risk categorization and illness evaluation capabilities.

However, existing evidence about the relationship between these four indices with the severity of CAS, especially in patients with ACS and CMM, remains limited. This study aims to examine the relationship between the non-HDL-C, RC, and their derived indices and the severity of CAS, as evaluated by the Gensini score, to offer more accurate biomarkers and clinical reference standards for early intervention and risk management in individuals with ACS and CMM.

## 2 Methods

### 2.1 Study design and participants

This retrospective study of 298 patients with ACS combined with CMM who were admitted to the Department of Cardiology at Qingdao Municipal Hospital from January 1, 2024, to July 31, 2024, and underwent coronary angiography (CAG). The selected cohort of ACS patients included unstable angina (UA), non-ST-elevation myocardial infarction (NSTEMI), and ST-elevation myocardial infarction (STEMI) (21). All participants were Chinese individuals aged 18 years and above. Exclusion criteria encompassed serious infections, malignant neoplasms, immunological or hematological diseases, and severe hepatic or renal illnesses. Patients with ACS combined with CMM were characterized as individuals having ACS in conjunction with one or more chronic conditions (hypertension, diabetes, and stroke). Hypertension is characterized by a systolic blood pressure of ≥140 mmHg and/or a diastolic blood pressure of ≥90 mmHg, or the present use of antihypertensive drugs (22). Diabetes is characterized by fasting blood glucose levels of ≥126 mg/dL or the individual is currently undergoing antidiabetic therapy (23). A stroke is characterized by a self-reported history of cerebrovascular disease.

The protocol was approved by the Ethics Committee of Qingdao Municipal Hospital (Approval Number: 2025-KY-154) and was free from the necessity of informed consent. The study complied with the Declaration of Helsinki.

### 2.2 Data collection

Through the review of electronic medical records, the subsequent general clinical data were gathered: age, gender, body mass index (BMI), smoking status, alcohol consumption status, history of statin use, history of percutaneous coronary intervention (PCI), history of UA, history of NSTEMI, history of STEMI, history of hypertension, history of diabetes, and history of stroke. Biochemical markers, including high-sensitivity C-reactive protein (hsCRP), fasting blood glucose, TC, triglycerides (TG), HDL-C, LDL-C, and apolipoprotein B (Apo B), were acquired via blood laboratory analyses. All blood samples were fasting venous specimens obtained within 24 hours of admission.

### 2.3 Definitions of non-HDL-C, NHHR, RC, and RCII

Non-HDL-C was determined using the formula: non-HDL-C (mg/dL) = TC - HDL-C. NHHR was computed as: non-HDL-C (mg/dL) divided by HDL-C (mg/dL) (24). RC was computed using the formula: RC (mg/dL) = TC - (HDL-C + LDL-C). RCII was computed as: RCII = RC (mg/dL) * hsCRP (mg/dL) (25).

### 2.4 Gensini score

Invasive CAG was performed via percutaneous radial or femoral artery angiography and conducted by experienced interventional cardiologists. The Gensini score was computed based on the findings of CAG. The Gensini score was the cumulative total of the stenosis scores for each coronary artery lesion, each multiplied by its respective lesion site score coefficient (26). A higher score indicated a more severe CAS. Severe CAS was characterized by a Gensini score over 32 points (27). The precise calculation technique for the Gensini score is detailed in **Supplementary Table S1**.

### 2.5 Statistical analysis

The Shapiro-Wilk normality test was initially conducted on continuous variables, indicating that they were not normally distributed. Consequently, they were reported as the median (quartile 1, quartile 3). Categorical variables were presented as percentages (%). The study population was statistically characterized by the mild-to-moderate CAS group and the severe CAS group. Based on the criteria described in section 2.4, the severe CAS group included patients with a Gensini score over 32 points, while the mild-to-moderate CAS group included patients with a Gensini score of 32 points or less. Differences in baseline characteristics across groups were analyzed using the Kruskal-Wallis rank-sum test for continuous variables and the chi-square test or Fisher’s exact test for categorical variables.

To investigate the association between non-HDL-C, NHHR, RC, and RCII with CAS, the specified indices were categorized into three groups according to their mean values, and multivariate logistic regression models were employed to ascertain the odds ratios (ORs) and 95% confidence intervals (CIs). Three models were developed: model 1: non-adjusted; model 2: adjusted for age, gender, and BMI; model 3: adjusted for age, gender, BMI, smoking, drinking, statin use, history of PCI, NSTEMI, and STEMI. Given the issue of collinearity, we assessed the generalized variance inflation factor (GVIF) of the covariates to guarantee that only those with GVIF < 5 were incorporated into the model. Comprehensive data for the multicollinearity tests are provided in **Supplementary Tables S2–S5** in the **Supplementary Material**. To facilitate data analysis, the exposure variables were standardized to z-scores, indicating variations in effect size for each 1 standard deviation (SD) increase.

Restricted cubic spline (RCS) analysis was conducted to elucidate the nonlinear relationship between various indices and CAS. The adjustment covariates of the RCS model aligned with those of the logistic regression analysis model 3, and the likelihood ratio test was employed to evaluate the nonlinear relationship. In the presence of a nonlinear relationship, we employed a recursive method to identify potential inflection points and utilized a two-segment logistic regression model to characterize the associations on either side of the inflection point.

A receiver operating characteristic (ROC) curve analysis was conducted to evaluate the predictive efficacy of several indices in determining the likelihood of severe CAS, utilizing DeLong’s test to compare the area under the curve (AUC). Subgroup analyses were performed according to age, gender, BMI, and diabetes status. The stratification factors were considered as potential effect modifiers, and interaction terms were incorporated to examine heterogeneity in associations among subgroups. In the sensitivity analysis, LDL-C and Apo B, two traditional indices for atherosclerosis, were used as covariates in multivariate logistic regression models to ascertain the ORs and 95% CIs.

All analyses were performed using R software (version 4.3.0), and a two-sided *P* value < 0.05 was considered statistically significant.

## 3 Results

### 3.1 Baseline characteristics of participants categorized by the degree of CAS

This study involved 298 participants with ACS combined with CMM, all of whom were hospitalized in the Department of Cardiology at Qingdao Municipal Hospital between January 1, 2024, and July 31, 2024. The median age was 66 years, with 71.48% of participants being male. Of all patients, 150 (50.34%) had severe CAS, whereas 148 (49.66%) presented with mild-to-moderate CAS. In comparison to the mild-to-moderate CAS group, the severe CAS group had a higher proportion of males, higher levels of hsCRP, LDL-C, Apo B, non-HDL-C, NHHR, RC, RCII, and a higher prevalence of STEMI, while HDL-C level and prevalence of UA were lower. No significant differences were identified for the remaining characteristics. A comprehensive summary of the characteristics of the participants can be found in **Table 1**.

**Table 1:** Baseline characteristics of participants categorized by the degree of CAS.

Characteristics	Total	Mild-to-moderate CAS	Severe CAS	*P* value
	(n = 298)	(n = 148)	(n = 150)	
Age, years	66.00 (58.25, 72.00)	66.00 (59.00, 72.25)	65.00 (57.00, 72.00)	0.288
Male, n (%)	213 (71.48)	96 (64.86)	117 (78.00)	0.012
BMI, kg/m²	25.95 (24.47, 27.76)	25.95 (24.22, 28.15)	25.95 (24.66, 27.58)	0.986
hsCRP, mg/L	0.50 (0.50, 2.15)	0.50 (0.50, 1.02)	0.60 (0.50, 3.55)	0.001
FBG, mg/dL	107.55 (90.41, 139.41)	105.21 (89.37, 135.99)	109.17 (91.17, 142.02)	0.392
TG, mg/dL	112.05 (84.15, 160.10)	109.83 (83.26, 153.01)	122.68 (85.92, 165.19)	0.197
TC, mg/dL	148.50 (121.23, 186.20)	145.97 (112.43, 180.49)	151.01 (124.62, 190.64)	0.057
HDL-C, mg/dL	41.38 (34.90, 47.56)	43.12 (36.64, 49.11)	39.83 (34.42, 46.69)	0.015
LDL-C, mg/dL	91.46 (69.99, 120.55)	86.04 (66.32, 111.47)	94.94 (75.79, 126.26)	0.014
Apo B, g/L	0.76 (0.62, 0.94)	0.74 (0.59, 0.89)	0.76 (0.65, 1.03)	0.018
Smoking, n (%)				0.631
Now	84 (28.19)	38 (25.68)	46 (30.67)	
Former	25 (8.39)	13 (8.78)	12 (8.00)	
Never	189 (63.42)	97 (65.54)	92 (61.33)	
Drinking, n (%)				0.675
Now	49 (16.44)	25 (16.89)	24 (16.00)	
Former	9 (3.02)	3 (2.03)	6 (4.00)	
Never	240 (80.54)	120 (81.08)	120 (80.00)	
Statin use, n (%)	92 (30.87)	47 (31.76)	45 (30.00)	0.743
History of PCI, n (%)	83 (27.85)	35 (23.65)	48 (32.00)	0.108
Hypertension, n (%)	256 (85.91)	128 (86.49)	128 (85.33)	0.775
Diabetes, n (%)	136 (45.64)	61 (41.22)	75 (50.00)	0.128
Stroke, n (%)	57 (19.13)	31 (20.95)	26 (17.33)	0.428
UA, n (%)	246 (82.55)	132 (89.19)	114 (76.00)	0.003
NSTEMI, n (%)	34 (11.41)	14 (9.46)	20 (13.33)	0.293
STEMI, n (%)	18 (6.04)	2 (1.35)	16 (10.67)	<0.001
Gensini score	32.75 (15.00, 58.00)	15.00 (9.00, 23.00)	58.00 (45.00, 81.50)	<0.001
Non-HDL-C, mg/dL	105.57 (81.40, 142.30)	99.96 (76.28, 134.57)	112.15 (87.78, 148.11)	0.007
NHHR	2.58 (1.98, 3.24)	2.31 (1.82, 3.02)	2.80 (2.13, 3.48)	<0.001
RC, mg/dL	15.08 (10.05, 21.66)	13.72 (8.02, 19.82)	16.04 (11.99, 23.20)	0.003
RCII	1.09 (0.60, 3.37)	0.94 (0.50, 1.69)	1.31 (0.70, 5.95)	<0.001

CAS, coronary artery stenosis; BMI, body mass index; hsCRP, high-sensitivity C-reactive protein; FBG, fasting blood glucose; TG, triglycerides; TC, total cholesterol; HDL-C, high-density lipoprotein cholesterol; LDL-C, low-density lipoprotein cholesterol; Apo B, apolipoprotein B; PCI, percutaneous coronary intervention; UA, unstable angina; NSTEMI, non-ST-elevation myocardial infarction; STEMI, ST-elevation myocardial infarction; NHHR, non-high-density lipoprotein cholesterol to high-density lipoprotein cholesterol ratio; RC, remnant cholesterol; RCII, remnant cholesterol inflammatory index.

### 3.2 Logistic regression models of the relationship between non-HDL-C, NHHR, RC, and RCII and the degree of CAS

**Figure 1:**
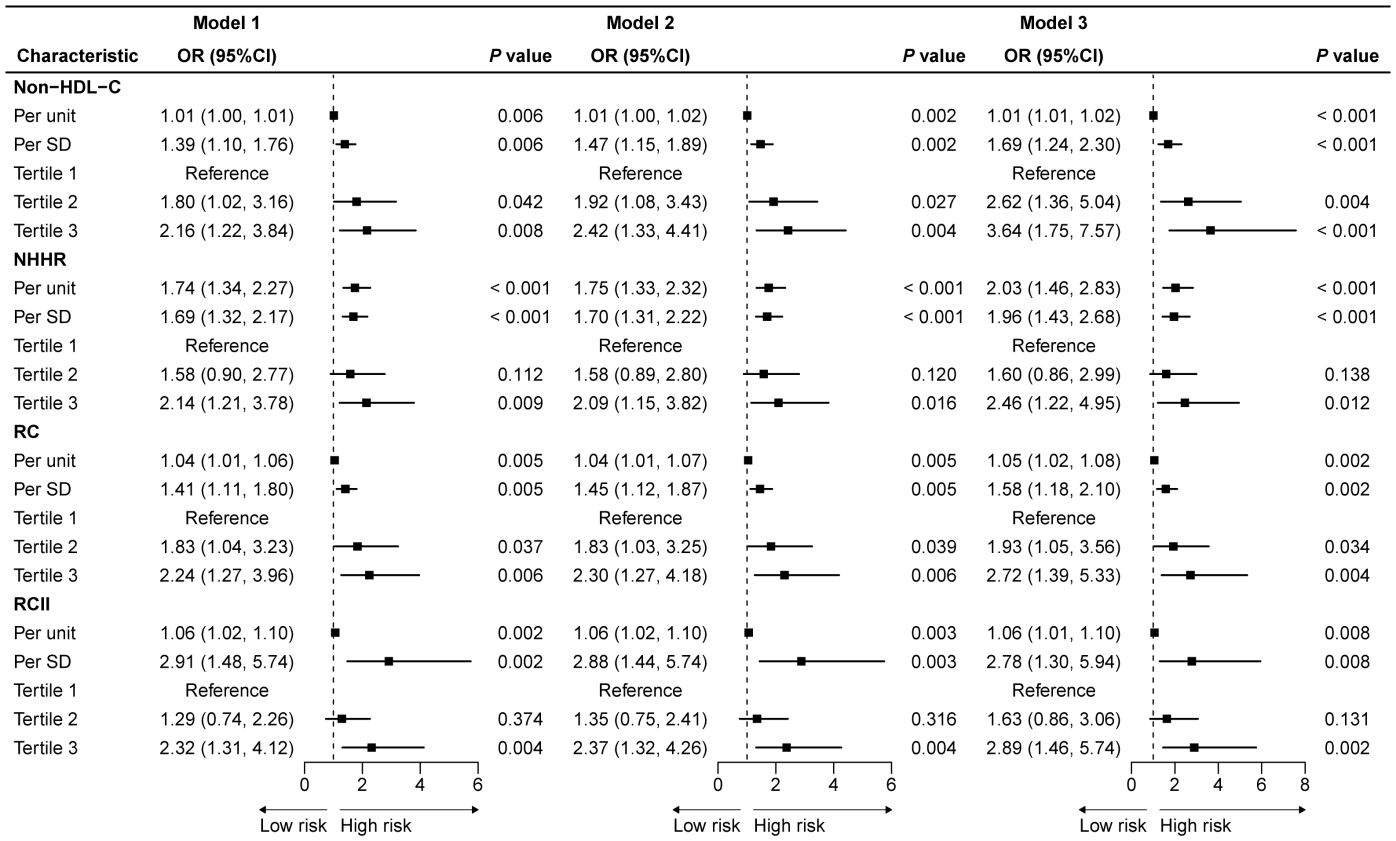
Logistic regression models examining the association between non-HDL-C, NHHR, RC, and RCII and the degree of CAS in patients with ACS combined with CMM. OR, odds ratio; CI, confidence interval; SD, standard deviation; non-HDL-C, non-high-density lipoprotein cholesterol; NHHR, non-high-density lipoprotein cholesterol to high-density lipoprotein cholesterol ratio; RC, remnant cholesterol; RCII, remnant cholesterol inflammatory index; CAS, coronary artery stenosis; ACS, acute coronary syndrome; CMM, cardiometabolic multimorbidity. The model was adjusted for age, gender, BMI, smoking, drinking, statin use, history of PCI, NSTEMI, and STEMI.

Multivariate logistic regression models were employed to examine the association between non-HDL-C, NHHR, RC, and RCII with CAS. Upon controlling for several covariates (model 3), the analysis of the aforementioned indices as continuous variables (each SD) revealed that all four indices exhibited a significant positive association with severe CAS. Of them, RCII had the strongest association with severe CAS (OR: 2.78, 95% CI 1.30-5.94), followed by NHHR (OR: 1.96, 95% CI 1.43-2.68). Thereafter, non-HDL-C, NHHR, RC, and RCII were categorized into three equal tertiles according to their mean values. The findings still indicated a substantial positive association with severe CAS. In comparison to the tertile 1 group, the association between non-HDL-C and severe CAS was the strongest in the tertile 3 group (OR: 3.64, 95% CI 1.75-7.57), followed by RCII (OR: 2.89, 95% CI 1.46-5.74). The detailed information of the logistic regression models is presented in **Figure 1**.

### 3.3 Nonlinear relationship of non-HDL-C, NHHR, RC, and RCII with the degree of CAS

**Figure 2:**
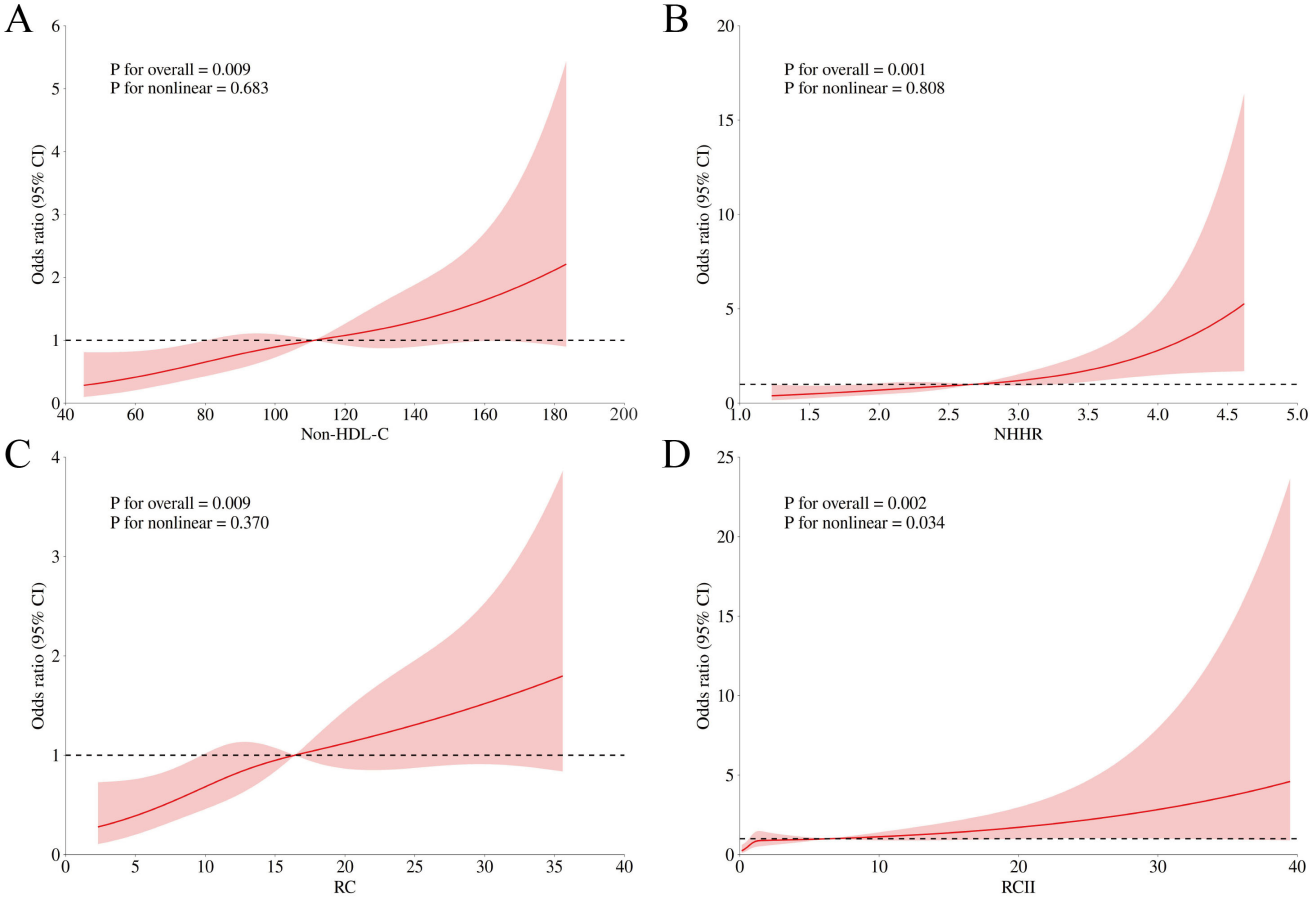
RCS analysis of the association between non-HDL-C, NHHR, RC, and RCII and the degree of CAS in patients with ACS combined with CMM. RCS, restricted cubic spline; non-HDL-C, non-high-density lipoprotein cholesterol; NHHR, non-high-density lipoprotein cholesterol to high-density lipoprotein cholesterol ratio; RC, remnant cholesterol; RCII, remnant cholesterol inflammatory index; CAS, coronary artery stenosis; ACS, acute coronary syndrome; CMM, cardiometabolic multimorbidity. **(A)** RCS analysis of the association between non-HDL-C and the degree of CAS. **(B)** RCS analysis of the association between NHHR and the degree of CAS. **(C)** RCS analysis of the association between RC and the degree of CAS. **(D)** RCS analysis of the association between RCII and the degree of CAS. The model was adjusted for age, gender, BMI, smoking, drinking, statin use, history of PCI, NSTEMI, and STEMI.

RCS models with multivariate logistic regression were employed to analyze the association between non-HDL-C, NHHR, RC, and RCII with CAS, to explore potential nonlinear trends. **Figure 2A** demonstrates an absence of a nonlinear relationship between non-HDL-C and severe CAS (*P* for nonlinear = 0.683). **Figure 2B** reveals no nonlinear relationship between NHHR and severe CAS (*P* for nonlinear = 0.808). **Figure 2C** indicates no nonlinear relationship between RC and severe CAS (*P* for nonlinear = 0.370), whereas **Figure 2D** shows a nonlinear relationship between RCII and severe CAS (*P* for nonlinear = 0.034). To further examine the association between RCII and severe CAS, we utilized segmented logistic regression and performed a threshold effect analysis, which yielded statistically significant findings in the likelihood ratio test (**Table 2**). A positive relationship existed between RCII and severe CAS (OR: 1.06, 95% CI 1.01-1.10). Specifically, when RCII was below 0.64, no association was observed between RCII and severe CAS. In contrast, when RCII surpassed 0.64, a positive association with severe CAS was noted (OR: 1.05, 95% CI 1.01-1.09).

**Table 2:** Threshold effect analysis of the association between RCII and the degree of CAS.

RCII	Adjusted OR (95%CI)	*P* value
Model 1: Fitting model by standard logistic regression	1.06 (1.01,1.10)	0.008
Model 2: Fitting model by two-piecewise logistic regression		
Inflection point	0.64	
<0.64	12.41 (0.62, 249.52)	0.100
≥0.64	1.05 (1.01, 1.09)	0.019
*P* for log likelihood ratio		0.021

OR, odds ratio; CI, confidence interval; RCII, remnant cholesterol inflammatory index; CAS, coronary artery stenosis.

The model was adjusted for age, gender, BMI, smoking, drinking, statin use, history of PCI, NSTEMI, and STEMI.

### 3.4 ROC analysis

**Figure 3:**
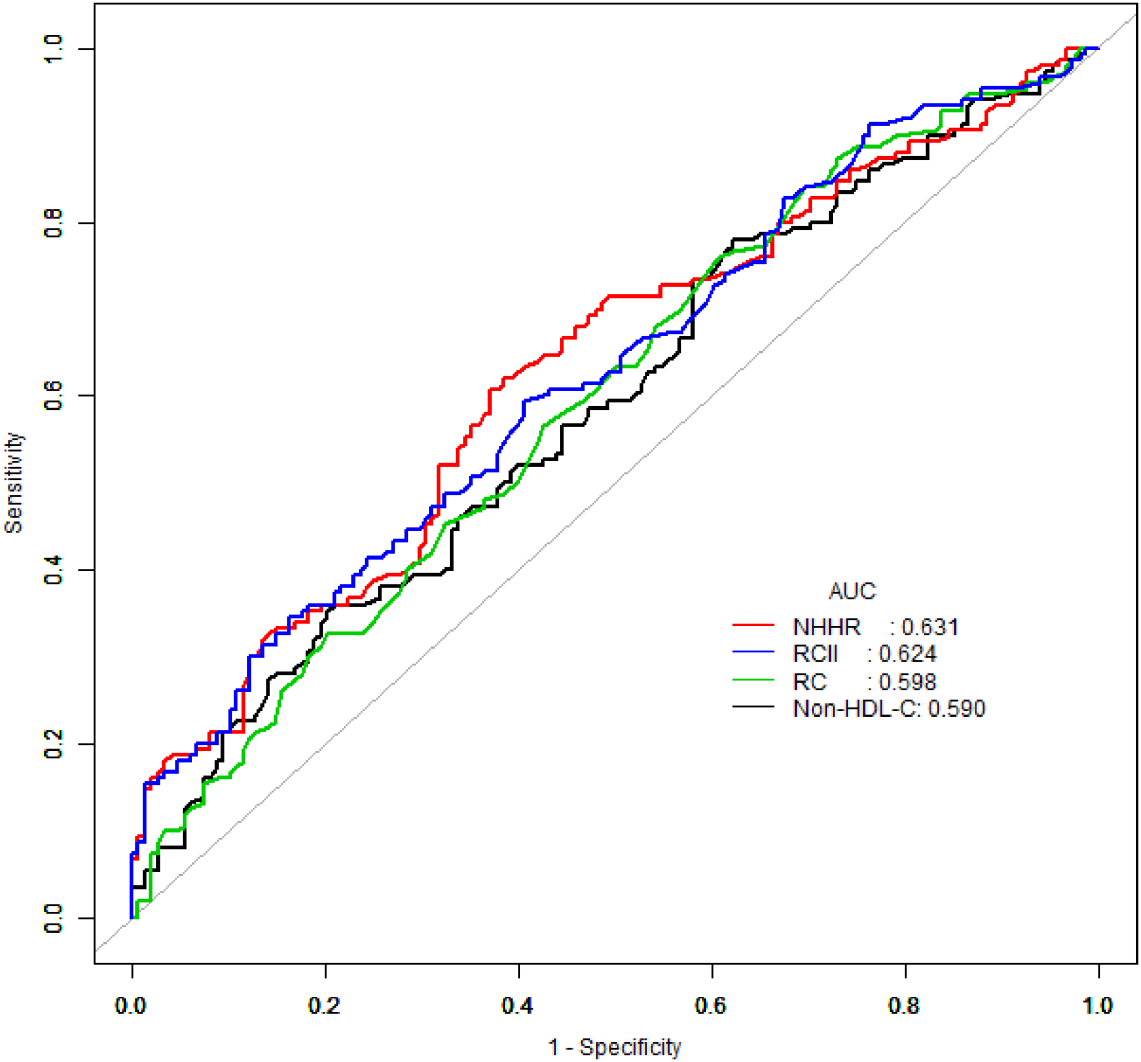
ROC curves analysis of the association between non-HDL-C, NHHR, RC, and RCII and severe CAS in patients with ACS combined with CMM. ROC curve, receiver operating characteristic curve; non-HDL-C, non-high-density lipoprotein cholesterol; NHHR, non-high-density lipoprotein cholesterol to high-density lipoprotein cholesterol ratio; RC, remnant cholesterol; RCII, remnant cholesterol inflammatory index; CAS, coronary artery stenosis; ACS, acute coronary syndrome; CMM, cardiometabolic multimorbidity.

ROC analysis was conducted to evaluate the predictive ability of non-HDL-C, NHHR, RC, and RCII for severe CAS, with the corresponding AUC values calculated (**Figure 3**). The findings indicated that NHHR exhibited the highest AUC value of 0.631, followed by RCII with an AUC value of 0.624. However, the difference between the two was not statistically significant (*P* = 0.804, Delong’s test). The detailed outcomes of Delong’s test are presented in **Supplementary Table S6**.

### 3.5 Subgroup analysis and sensitivity analysis

Subgroup analyses were conducted based on age, gender, BMI, and diabetes status after adjusting for covariates (**Table 3**). The findings indicated that the relationship between non-HDL-C, NHHR, RC, and RCII with CAS was stable across all subgroups (*P* interaction > 0.05). In the sensitivity analysis, LDL-C and Apo B, two traditional indices for atherosclerosis, were used as covariates in model 3 for multivariate logistic regression (**Supplementary Table S7**). The findings indicated that when these measures were analyzed as continuous variables (each SD), NHHR and RCII remained strongly positively associated with severe CAS. The most significant association was identified between RCII and severe CAS (OR: 2.47, 95% CI 1.15-5.31), followed by NHHR (OR: 2.04, 95% CI 1.30-3.18).

**Table 3:** Subgroup analysis of the association between non-HDL-C, NHHR, RC, and RCII and the degree of CAS.

Variables	Severe CAS		
	OR(95% CI)	*P* value	*P* interaction
Non-HDL-C per SD			
Age, years			0.698
≤65	1.58 (1.05, 2.37)	0.028	
>65	1.75 (1.18, 2.60)	0.006	
Gender			0.078
Male	2.00 (1.36, 2.94)	<0.001	
Female	1.10 (0.63, 1.90)	0.743	
BMI, kg/m2			0.752
<25	1.79 (1.09, 2.96)	0.023	
≥25	1.63 (1.14, 2.34)	0.007	
Diabetes			0.484
Yes	1.46 (0.99, 2.15)	0.055	
No	1.73 (1.13, 2.77)	0.013	
NHHR per SD			
Age, years			0.535
≤65	1.75 (1.15, 2.65)	0.009	
>65	2.08 (1.38, 3.15)	<0.001	
Gender			0.941
Male	1.96 (1.36, 2.82)	<0.001	
Female	1.91 (1.04, 3.49)	0.037	
BMI, kg/m2			0.359
<25	2.38 (1.38, 4.10)	0.002	
≥25	1.78 (1.23, 2.57)	0.002	
Diabetes			0.535
Yes	1.74 (1.14, 2.66)	0.010	
No	2.08 (1.35, 3.23)	0.001	
RC per SD			
Age, years			0.361
≤65	1.37 (0.94, 2.01)	0.101	
>65	1.76 (1.18, 2.61)	0.005	
Gender			0.234
Male	1.77 (1.23, 2.55)	0.002	
Female	1.23 (0.76, 2.00)	0.403	
BMI, kg/m2			0.562
<25	1.77 (1.09, 2.85)	0.020	
≥25	1.49 (1.06, 2.11)	0.021	
Diabetes			0.882
Yes	1.49 (1.00, 2.23)	0.049	
No	1.56 (1.03, 2.35)	0.035	
RCII per SD			
Age, years			0.473
≤65	4.47 (0.96, 20.86)	0.057	
>65	2.45 (1.09, 5.49)	0.029	
Gender			0.640
Male	2.61 (1.19, 5.72)	0.016	
Female	4.30 (0.58, 31.63)	0.152	
BMI, kg/m2			0.804
<25	3.08 (0.87, 10.84)	0.080	
≥25	2.53 (1.00, 6.38)	0.050	
Diabetes			0.984
Yes	2.53 (0.95, 6.71)	0.063	
No	2.49 (0.83, 7.50)	0.105	

OR, odds ratio; CI, confidence interval; SD, standard deviation; CAS, coronary artery stenosis; non-HDL-C, non-high-density lipoprotein cholesterol; NHHR, non-high-density lipoprotein cholesterol to high-density lipoprotein cholesterol ratio; RC, remnant cholesterol; RCII, remnant cholesterol inflammatory index; BMI, body mass index.

The model was adjusted for age, gender, BMI, smoking, drinking, statin use, history of PCI, NSTEMI, and STEMI. The variable for subgroup stratification was left out.

## 4 Discussion

This study revealed the relationship among non-HDL-C, NHHR, RC, and RCII with the degree of CAS in patients with ACS combined with CMM. The findings indicated that non-HDL-C, NHHR, RC, and RCII were all strongly associated with severe CAS, with RCII and NHHR exhibiting stronger associations with CAS. A nonlinear relationship was detected between RCII and severe CAS, with threshold effect analysis pinpointing 0.64 as the inflection point. ROC analysis demonstrated that NHHR possessed the highest predictive capability, followed by RCII. The research highlighted the strong association between RCII and NHHR, and CAS in individuals with ACS and CMM.

Coronary artery disease, hypertension, diabetes, and stroke are characteristic of CMM, a prevalent pattern of multimorbidity now recognized (28, 29). CMM is not merely the aggregation of many diseases; instead, it represents a synergistic amplification effect arising from shared pathophysiological mechanisms, including chronic inflammation, insulin resistance, and oxidative stress, which together accelerate the progression of atherosclerosis. Insulin resistance results in increased very low-density lipoproteins secretion by the liver and the accumulation of triglyceride-rich particles in the bloodstream (30), while also stimulating the release of pro-inflammatory factors through the activation of inflammatory pathways like Nuclear factor kappa B (31). This chain reaction induces inflammatory responses and oxidative stress, ultimately causing myocardial damage and atherosclerosis (32, 33). The mechanical stress of hypertension causes alterations in vascular structure and function, resulting in heightened arterial stiffness, vascular inflammation, and endothelial dysfunction (34, 35). Furthermore, stroke may trigger systemic inflammatory and immunological responses (36, 37). This detrimental loop of comorbidities finally results in more severe coronary artery lesions. Conventional lipid management approaches frequently exhibit limits in complicated individuals, underscoring the pressing necessity to discover novel indices that more thoroughly represent diseases associated with CMM.

This study found a positive relationship between non-HDL-C and the degree of CAS in individuals with ACS and CMM. Similarly, Zhang et al. discovered that non-HDL-C exhibited a positive relationship with the degree of CAS, demonstrating superiority over LDL-C (38). Non-HDL-C encompasses all plasma lipoproteins excluding HDL. Alongside LDL-C, several other components of non-HDL-C, including TRL, TRL remnants, and lipoprotein a, have atherogenic effects (39, 40). This may explain why non-HDL-C exhibits a greater association with CAS compared to LDL-C. This study additionally revealed that NHHR had the most positive association and predictive efficacy concerning CAS. The study by Yang et al. demonstrated that NHHR exhibited a positive association with CAS and provided superior prediction efficiency compared to conventional lipid indices (41). The research conducted by Gao et al. revealed that NHHR also exhibited a positive association with CAS in patients with STEMI (42). These are similar to the findings of this investigation. Ding et al. identified a nonlinear association between NHHR and coronary heart disease, which contradicts the findings of this study (43). The primary distinction may reside in the study’s population, which concentrated on ACS combined with CMM, whereas the outcome was characterized by the degree of CAS. NHHR assesses the overall burden of atherogenic lipoproteins (non-HDL-C) while also including beneficial lipoproteins (HDL-C). HDL provides cardiovascular protection by facilitating cholesterol reverse transport, inhibiting oxidative stress, and exerting anti-inflammatory effects (44). Within the framework of CMM, HDL function frequently transitions from protective to dysfunctional, exhibiting significantly diminished cholesterol reverse transport ability and anti-inflammatory characteristics (45, 46). Consequently, in this condition, an increase in NHHR levels signifies not only a rise in non-HDL-C associated with atherosclerosis but also indicates a diminished function of protective HDL-C. The twofold imbalance renders NHHR a superior lipid metabolic composite index for evaluating the probability of CAS in individuals with ACS and CMM.

RC is defined as the cholesterol content of residual lipoproteins produced during vascular remodeling from TRL. These lipoproteins are extensively absorbed by macrophages, effectively forming foam cells that advance atherosclerosis (47). Increased RC levels also represent one of the atherogenic lipid characteristics in diabetes (48). This study illustrates that RC is positively associated with the degree of CAS in patients with ACS and CMM. This discovery corresponds with prior research (49, 50). The paramount discovery pertains to the outstanding efficacy of its derived metric, RCII. Results demonstrate that RCII has the highest association with CAS, exhibiting a nonlinear relationship with an inflection point at 0.64. Recent research by Yu et al. established a notable association between RCII and negative outcomes in acute ischemic stroke (20). Wang et al. identified a J-shaped relationship between LnRCII and cardiovascular mortality in middle-aged and elderly populations (51). Notwithstanding variations in research populations and outcomes, these studies collectively affirm the strong association between RCII and cardiovascular as well as cerebrovascular disorders. Elevated levels of hsCRP, a characteristic inflammatory marker in the atherosclerotic process, have long been shown to be associated with cardiovascular risk similar to that of elevated LDL-C levels (52). Poole et al. established that increased CRP levels were associated with CMM outcomes following 14 years of follow-up (53). Masood et al. identified a significant relationship between hsCRP levels and the severity of CAS as evaluated by the Gensini score (54). Furthermore, Zhang et al. further established that hsCRP synergistically amplifies RC’s prognostic capability for coronary lesion severity (55). A key finding of this study is that, despite lower median hsCRP levels overall, RCII had a stronger association and greater predictive ability for severe CAS than RC alone. We contend that this is not solely a mathematical consequence but rather reveals a significant synergistic interaction between lipid metabolism and inflammatory state. This indicates that in the pathogenic environment of CMM, even subclinical or mild inflammation might function as an ‘amplifier’, markedly increasing the atherogenic potential of RC.

The core of ACS is rooted in plaque instability and rupture, a process intricately linked to vigorous inflammatory responses and the accumulation of TRL (56). Therefore, RCII, as a quantitative measure of the relationship between lipid accumulation and inflammatory severity, demonstrates a markedly elevated value in the acute pathophysiological context of ACS. Our findings indicate that RC and RCII may function not only as indicators of overall atherosclerotic burden but also as possible biomarkers for evaluating its instability. This has considerable clinical ramifications for patient risk assessment and treatment decisions.

This study used a single-center retrospective design, which inherently poses issues of selection bias and information bias. Secondly, although we diligently gathered and documented the principal confounding variable, statin use, data regarding other medications (such as non-statin lipid-lowering agents, antihypertensive medications, and antidiabetic drugs) were inadequate and inconsistent across the sources, leading to their exclusion. Moreover, the pathophysiology of ACS transcends mere anatomical stenosis, significantly implicating blood cell dynamics and endothelial dysfunction, especially in individuals with diabetes. The Gensini score, as an anatomical metric, inadequately encompasses the functional pathological features of ACS. The underlying mechanisms require clarification via future prospective studies.

## 5 Conclusions

Non-HDL-C, NHHR, RC, and RCII all showed a strong association with CAS in patients with ACS combined with CMM. RCII exhibited a nonlinear association with severe CAS, featuring an inflection point at 0.64. Incorporating RCII into future clinical practice will enable physicians to formulate more precise prognoses and improve medical care for patients with ACS complicated by CMM.

## Data Availability

The original contributions presented in the study are included in the article/**Supplementary Material**. Further inquiries can be directed to the corresponding authors.
