# Supplementary material for: The association of non-HDL-C, NHHR, RC, and RCII with coronary artery stenosis severity in patients with acute coronary syndrome combined with cardiometabolic multimorbidity
Source: Front Endocrinol (Lausanne). 2025 Dec 4;16:1701364. doi: 10.3389/fendo.2025.1701364 (PMC12711502; doi:10.3389/fendo.2025.1701364)
Supplement: Supplementary file 1 [file Table1.docx]

**Table S1. Gensini score rating rules**

| **Degree of coronary stenosis** | **Value of a score** | **Diseased vessel** | **Coefficient** |
| --- | --- | --- | --- |
| ≤25% | 1 | Left main coronary artery | 5 |
| 26-50% | 2 | Left anterior descending artery | Proximal segment (2.5) |
| 51-75% | 4 |  | Midsegment (1.5) |
| 76-90% | 8 |  | Distal segment (1) |
| 91-99% | 16 | First diagonal | 1 |
| 100% | 32 | Second diagonal | 1 |
|  |  | Left circumflex artery | Proximal segment (2.5) |
|  |  |  | Mid-distal segment (1) |
|  |  | Obtuse marginal artery | 1 |
|  |  | Posterior descending artery | 1 |
|  |  | Posterolateral | 0.5 |
|  |  | Right coronary artery | Proximal segment (1) |
|  |  |  | Midsegment (1) |
|  |  |  | Distal segment (1) |
|  |  | Posterior descending artery | 1 |

Single vessel score = coefficient of diseased vessel * score corresponding to the degree of stenosis of the diseased vessel

Gensini score = sum of all vessel scores

**Table S2.** **Covariance analysis of non-HDL-C and other variables**

|  | **GVIF** | **Df** | **GVIF^1/2Df^** |
| --- | --- | --- | --- |
| Non-HDL-C | 1.52 | 1 | 1.23 |
| Age | 1.29 | 1 | 1.14 |
| Gender | 1.43 | 1 | 1.19 |
| BMI | 1.13 | 1 | 1.06 |
| Smoking | 2.12 | 2 | 1.21 |
| Drinking | 1.69 | 2 | 1.14 |
| Statin use | 1.26 | 1 | 1.12 |
| History of PCI | 1.36 | 1 | 1.17 |
| NSTEMI | 1.08 | 1 | 1.04 |
| STEMI | 1.15 | 1 | 1.07 |

Abbreviations: GVIF, generalized variance inflation factor; Df, degree of freedom; BMI, body mass index; PCI, percutaneous coronary intervention; NSTEMI, non-ST-elevation myocardial infarction; STEMI, ST-elevation myocardial infarction.

**Table S3.** **Covariance analysis of NHHR and other variables**

|  | **GVIF** | **Df** | **GVIF^1/2Df^** |
| --- | --- | --- | --- |
| NHHR | 1.45 | 1 | 1.20 |
| Age | 1.30 | 1 | 1.14 |
| Gender | 1.36 | 1 | 1.17 |
| BMI | 1.13 | 1 | 1.06 |
| Smoking | 2.16 | 2 | 1.21 |
| Drinking | 1.69 | 2 | 1.14 |
| Statin use | 1.27 | 1 | 1.13 |
| History of PCI | 1.30 | 1 | 1.14 |
| NSTEMI | 1.08 | 1 | 1.04 |
| STEMI | 1.12 | 1 | 1.06 |

Abbreviations: GVIF, generalized variance inflation factor; Df, degree of freedom; NHHR, non-high-density lipoprotein cholesterol to high-density lipoprotein cholesterol ratio; BMI, body mass index; PCI, percutaneous coronary intervention; NSTEMI, non-ST-elevation myocardial infarction; STEMI, ST-elevation myocardial infarction.

**Table S4.** **Covariance analysis of RC and other variables**

|  | **GVIF** | **Df** | **GVIF^1/2Df^** |
| --- | --- | --- | --- |
| RC | 1.27 | 1 | 1.13 |
| Age | 1.29 | 1 | 1.14 |
| Gender | 1.38 | 1 | 1.18 |
| BMI | 1.12 | 1 | 1.06 |
| Smoking | 2.15 | 2 | 1.21 |
| Drinking | 1.68 | 2 | 1.14 |
| Statin use | 1.24 | 1 | 1.11 |
| History of PCI | 1.29 | 1 | 1.13 |
| NSTEMI | 1.06 | 1 | 1.03 |
| STEMI | 1.10 | 1 | 1.05 |

Abbreviations: GVIF, generalized variance inflation factor; Df, degree of freedom; RC, remnant cholesterol; BMI, body mass index; PCI, percutaneous coronary intervention; NSTEMI, non-ST-elevation myocardial infarction; STEMI, ST-elevation myocardial infarction.

**Table S5.** **Covariance analysis of RCII and other variables**

|  | **GVIF** | **Df** | **GVIF^1/2Df^** |
| --- | --- | --- | --- |
| RCII | 1.23 | 1 | 1.11 |
| Age | 1.24 | 1 | 1.11 |
| Gender | 1.37 | 1 | 1.17 |
| BMI | 1.12 | 1 | 1.06 |
| Smoking | 2.16 | 2 | 1.21 |
| Drinking | 1.70 | 2 | 1.14 |
| Statin use | 1.21 | 1 | 1.10 |
| History of PCI | 1.25 | 1 | 1.12 |
| NSTEMI | 1.11 | 1 | 1.05 |
| STEMI | 1.18 | 1 | 1.09 |

Abbreviations: GVIF, generalized variance inflation factor; Df, degree of freedom; RCII, remnant cholesterol inflammatory index; BMI, body mass index; PCI, percutaneous coronary intervention; NSTEMI, non-ST-elevation myocardial infarction; STEMI, ST-elevation myocardial infarction.

**Table S6. Paired comparison of ROC curves (DeLong's test)**

|  | **Difference of AUC** | ***P* value** |
| --- | --- | --- |
| Non-HDL-C vs. NHHR | 0.041 | 0.044 |
| Non-HDL-C vs. RC | 0.008 | 0.697 |
| Non-HDL-C vs. RCII | 0.034 | 0.249 |
| NHHR vs. RC | 0.033 | 0.106 |
| NHHR vs. RCII | 0.007 | 0.804 |
| RC vs. RCII | 0.026 | 0.335 |

Abbreviations: ROC, receiver operating characteristic; AUC, area under the curve; non-HDL-C, non-high-density lipoprotein cholesterol; NHHR, non-high-density lipoprotein cholesterol to high-density lipoprotein cholesterol ratio; RC, remnant cholesterol; RCII, remnant cholesterol inflammatory index.

**Table S7.** **Sensitivity analysis**

|  | **Model 1** |  | **Model 2** |  | **Model 3** |  |
| --- | --- | --- | --- | --- | --- | --- |
| **Per SD** | **OR(95% CI)** | ***P* value** | **OR(95% CI)** | ***P* value** | **OR(95% CI)** | ***P* value** |
| Non-HDL-C | 1.39 (1.10, 1.76) | 0.006 | 1.47 (1.15, 1.89) | 0.002 | 3.71 (0.89, 15.50) | 0.073 |
| NHHR | 1.69 (1.32, 2.17) | < 0.001 | 1.70 (1.31, 2.22) | < 0.001 | 2.04 (1.30, 3.18) | 0.002 |
| RC | 1.41 (1.11, 1.80) | 0.005 | 1.45 (1.12, 1.87) | 0.005 | 1.36 (0.97, 1.91) | 0.075 |
| RCII | 2.91 (1.48, 5.74) | 0.002 | 2.88 (1.44, 5.74) | 0.003 | 2.47 (1.15, 5.31) | 0.021 |

Abbreviations: OR, odds ratio; CI, confidence interval; SD, standard deviation; non-HDL-C, non-high-density lipoprotein cholesterol; NHHR, non-high-density lipoprotein cholesterol to high-density lipoprotein cholesterol ratio; RC, remnant cholesterol; RCII, remnant cholesterol inflammatory index.

Model 1: non-adjusted.

Model 2: adjusted for age, gender, and BMI.

Model 3: adjusted for age, gender, BMI, LDL-C, Apo B, smoking, drinking, statin use, history of PCI, NSTEMI, and STEMI.
